# Cellular Crosstalk Within Magnetically Functionalised Hydrogel-Composite Scaffolds for Enhanced Vascularisation and Bone Repair

**DOI:** 10.3390/gels12040315

**Published:** 2026-04-07

**Authors:** Jingyi Xue, Neelam Gurav, Sanjukta Deb

**Affiliations:** Faculty of Dentistry, Oral and Craniofacial Sciences, King’s College London, London SE1 9RT, UK; j.xue@qmul.ac.uk (J.X.);

**Keywords:** biomimetic scaffolds, hydrogel composites, magnetic nanoparticles, bone tissue engineering, angiogenesis, oral and maxillofacial bone regeneration

## Abstract

Repairing maxillofacial bone defects remains a major clinical challenge due to inadequate vascularisation and poor integration with host tissue. While bioactive scaffolds have shown promise in supporting osteogenesis and angiogenesis, achieving robust and synchronised dual regenerative outcomes is still elusive. This study presents a multifunctional, cell-free magnetic hydrogel platform designed to biomimetically coordinate osteogenic and angiogenic processes for effective maxillofacial bone regeneration. The composite poly(vinyl alcohol)-vaterite (PVA-Vat) hydrogel scaffold incorporates tuneable magnetic nanoparticles (MNPs) composed of single-domain superparamagnetic iron oxide (Fe_3_O_4_). By harnessing magneto-mechanical cues to orchestrate bilateral communication between human bone mesenchymal stem cells and endothelial cells, this platform provides a deeper mechanistic understanding of coupled tissue regeneration and delivers superior dual-regenerative performance for maxillofacial bone repair. Under magnetic stimulation, a coculture system demonstrated strong osteogenesis-angiogenesis coupling mediated by reciprocal VEGFA-BMP2 signalling. This reciprocal crosstalk was evidenced by a synergistic amplification of VEGFA and BMP2 expression in coculture compared to monocultures, where MNP-stimulated osteoprogenitors secreted VEGFA to drive endothelial capillary-like network formation, while endothelial cells reciprocally enhanced endogenous BMP2 levels to accelerate osteoblastic mineralisation. These findings establish MNP-integrated hydrogels as a cell-free, multifunctional platform capable of synchronising dual regenerative pathways, offering a biomimetic strategy to overcome vascularisation and integration barriers in maxillofacial bone repair.

## 1. Introduction

Reconstruction of maxillofacial bone defects remains a significant clinical challenge, as successful repair depends on rapid and stable vascularisation to maintain cell viability and preserve facial structure and function [1]. While autologous bone grafts are the benchmark, they are constrained by donor site morbidity and limited tissue availability [2]. Emerging tissue engineering strategies seek to mimic the native extracellular matrix (ECM) with biomimetic scaffolds that deliver cells and bioactive cues capable of guiding osteogenic and angiogenic processes [3]. Nevertheless, uncontrolled release of exogenous molecules and immune responses to transplanted cells often yield variable outcomes in vivo [4].

Hydrogels play multiple roles to help support and regenerate bone tissue. They offer significant advantages in forming a matrix for bone tissue engineering because their three-dimensional porous architecture closely mimics the native ECM, providing an ideal environment for cell attachment, proliferation, and tissue regeneration [5]. The gel polymeric network consists of crosslinked hydrophilic chains that provide high affinity to moisture, enabling absorption and retention of large amounts of biological fluids. 3D, water-swollen hydrogel networks in tissue engineering can also serve as cell carriers, growth factor depots, and templates for mineral deposition, often used alone or as part of composite scaffolds [6,7]. Polymers such as alginate, gelatine, collagen, hyaluronic acid, chitosan and poly(vinyl alcohol) (PVA) form biocompatible hydrogels and thus are attractive in tissue engineering applications.

PVA is a water-soluble polymer that can be formed into a hydrogel through physical crosslinking using a freeze-thawing process that not only confers a crosslinked matrix but also enhances porosity [8]. Porosity in a hydrogel composite for bone tissue regeneration is important since it allows vascular infiltration and nutrient and oxygen transport, thus enhancing osteogenesis and vascularisation. Furthermore, PVA can be engineered to form hydrogels with variable gradient mechanical stiffness and shape adaptive scaffolds [9].

A next generation of three-dimensional (3D) porous scaffolds has emerged that relies on endogenous cell recruitment, enabling in situ osteogenesis and angiogenesis through intrinsic osteoinductive cues [10,11]. Deb et al. [12] previously reported hydrogel composites using PVA as the matrix, demonstrating improved clinical handling, easier manipulation, greater water absorption, elastomeric behaviour, and enhanced osteoconductive performance. PVA with a MW of 145 kDA and a hydrolysis degree of ≥98% undergoes adequate hydrolytic degradation and has been shown to be effectively cleared renally in studies involving mice and rabbits [13,14]. Recently, we introduced a class of magnetic poly(vinyl alcohol)-vaterite (PVA-Vat) scaffolds integrated with superparamagnetic iron oxide nanoparticles (MNPs) [15]. These PVA-Vat-MNP hydrogel composites feature an interconnected macroporous architecture, high water uptake, and a compressive strength (12.88–21.22 MPa) and modulus (209.13–284.50 MPa) that effectively match or exceed mandibular trabecular bone (0.2–10.4 MPa and 3.5–125.6 MPa, respectively) [16], while their surface chemistry facilitates apatite mineralisation [15].

Maxillofacial bone repair primarily occurs through intramembranous ossification, a process that requires finely coordinated osteogenesis and angiogenesis; endothelial cells form a vascular template that delivers oxygen, nutrients, and osteoprogenitors to the defect site [17]. To enhance this natural healing cascade, advanced tissue engineering strategies incorporate scaffolds embedded with single-domain superparamagnetic iron oxide nanoparticles, recognised for their biocompatibility. Even without an external magnetic field, these nanoparticles generate local magneto-mechanical forces that exploit the inherent mechanosensitivity of osteogenic and endothelial cells [18,19]. This process of converting physical cues into biochemical signals, known as “mechanotransduction”, exerts nanoscale forces on the cell membrane, either directly or through the ECM, and activates specific mechanosensitive ion channels like Piezo1 and voltage-gated calcium channels, leading to calcium ion influx, cytoskeletal reorganisation, and the initiation of downstream signalling pathways crucial for cell proliferation, differentiation, and tissue formation [20,21]. The US FDA has already approved pulsed electromagnetic fields (PEMFs) as a safe and effective treatment for non-union of bone, where cell membrane receptors are identified as the site of action of electromagnetic stimulation, facilitating ECM synthesis for bone and cartilage repair [22,23]. This provides a mechanistic rationale for the application of MNPs in hydrogel scaffolds to stimulate bone regeneration in bone tissue engineering.

We present a comprehensive study into how the graded integration of MNPs within hydrogel composites of PVA-Vat scaffolds dynamically influences osteogenic and angiogenic pathways, independent of systemic variables. Beyond evaluating cellular responses, we uncover the mechanistic interplay between cocultured endothelial and osteoprogenitor cells, demonstrating how scaffold architecture and magnetic cues synergistically drive osteogenesis-angiogenesis coupling. This work redefines the scaffold as not merely a passive substrate but as an active, bio-instructive platform capable of orchestrating complex tissue regeneration processes.

Establishing this foundational dataset provides the scientific basis needed to mitigate risk and inform future progression towards more complex in vivo animal studies, thereby reinforcing the scaffold’s potential as a cell-free, multifunctional platform for maxillofacial bone regeneration.

## 2. Results and Discussion

### 2.1. PVA-Vat-MNP Scaffolds

**Figure 1:**
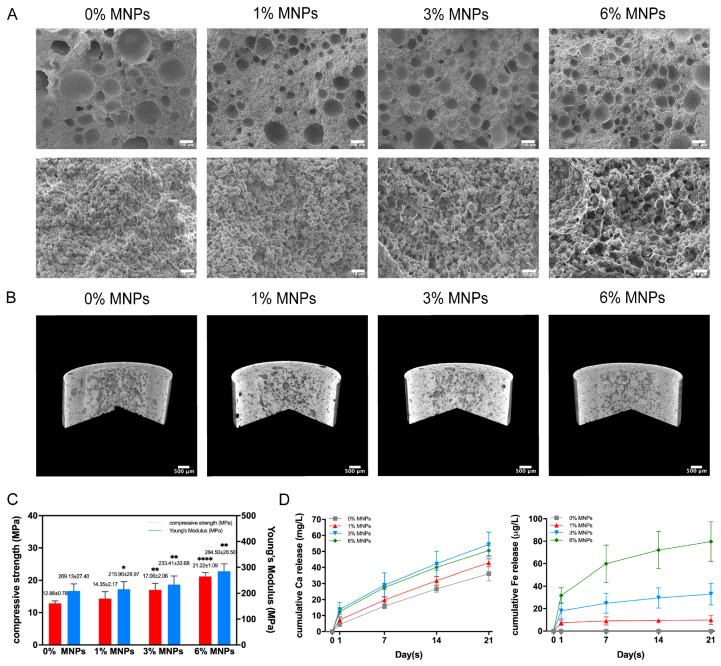
(**A**) SEM images and (**B**) μCT images of PVA-Vat-MNP scaffolds, showing the 3D interconnected porous structure with spherical vaterite particles uniformly distributed within the crosslinked PVA matrix [15]. Scale bars in A represent 100 μm and 10 μm; in B, they represent 500 μm. (**C**) Compressive strength and Young’s modulus of scaffolds 0% MNPs, 1% MNPs, 3% MNPs and 6% MNPs. Data are represented as mean ± SD, *n* = 6, * indicating statistical significance compared to the 0% MNP control group (* *p* < 0.05, ** *p* ≤ 0.01, and **** *p* ≤ 0.0001) [15]. (**D**) Cumulative ion release of PVA-Vat-MNP scaffolds in deionised water for up to 21 days, *n* =3.

The three-dimensional PVA-Vat-MNP scaffolds feature an interconnected network (Figure 1A,B) that provides structural integrity to support cell adhesion and maxillofacial bone regeneration, which has been published in our previous studies [15]. The magnetic scaffolds showed increased strength with higher MNP levels (Figure 1C), attributed to the strong interactions between MNPs and PVA through synergetic complexation, hydrogen bonding and physical crosslinking [24]. These interactions provide sufficient mechanical support to facilitate cell adhesion and bone formation. The scaffolds exhibited an initial burst release of calcium ions within the first 24 h of immersion in deionised water (Figure 1D). Such an early release is known to promote apatite formation and biomineralisation in physiological conditions while also driving the controlled biodegradation of the composite scaffolds, which is essential for subsequent tissue ingrowth and new bone formation [25]. A sustained 21-day release followed, with higher MNP content improving release efficiency (*p* < 0.0001). Similarly, all magnetic scaffolds showed a burst release of iron ions in the early stages, and the higher concentration of MNPs facilitated faster release kinetics, resulting in significantly greater cumulative iron release: 2.8% ± 0.9%, 5.0% ± 0.7%, and 9.2% ± 2.0% of the total MNP content for the 1%, 3%, and 6% MNPs scaffolds, respectively. Despite the initial burst, all scaffolds demonstrated a steady and sustained iron release, with less than 10% of total MNP content released over 21 days. This controlled profile is crucial for striking a balance between stimulating bone regeneration and mitigating risks of cytotoxicity.

### 2.2. Osteogenic Differentiation on PVA-Vat-MNP Scaffolds

**Figure 2:**
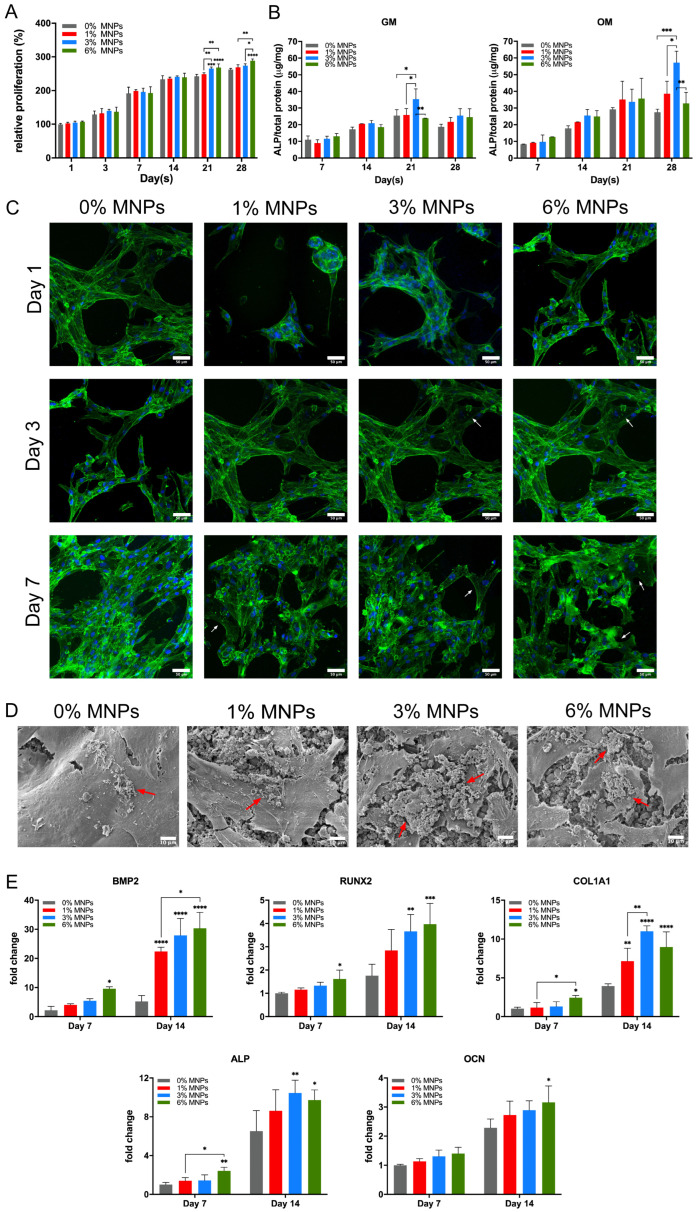
(**A**) Relative cell proliferation rate of hBMSCs cultured on PVA-Vat-MNP scaffolds for up to 28 days, normalised to cells on 0% MNP scaffolds at day 1. (**B**) ALP activity of hBMSCs cultured on scaffolds in GM or OM for up to 28 days. (**C**) Actin cytoskeleton staining of hBMSCs at days 1, 3 and 7 showing green-stained actin filaments and blue-stained nuclei; polygonal-shaped cells are indicated by white arrows. (**D**) SEM images of hBMSCs after 28 days in OM, revealing cauliflower-like mineralised nodules (red arrows) on the cell sheet surface. (**E**) Osteogenic gene expression of hBMSCs at days 7 and 14, normalised to gene expression on 0% MNP scaffolds at day 7. Scale bars in panel (**C**) represent 50 μm, and in panel (**D**) represent 10 μm. * *p* < 0.05, ** *p* ≤ 0.01, *** *p* ≤ 0.001, and **** *p* ≤ 0.0001.

hBMSC proliferation increased on all scaffolds, with higher MNP levels further enhancing growth (Figure 2A). At later culture stages, cell proliferation was markedly higher on scaffolds containing the greater concentrations of MNPs, with the 6% MNP scaffolds showing the highest proliferation rate. Notably, proliferation on 3% MNP scaffolds plateaued after 21 days, with no significant increase observed from day 21 to day 28. This indicated a shift from proliferation to differentiation, as supported by previous findings [26]. Consistently, cells seeded on 3% MNP scaffolds displayed significantly elevated ALP activity in GM (Figure 2B), which further increased in OM, peaking at day 28. This indicates enhanced osteoblastic differentiation driven by the synergistic effects of MNPs and the osteoinductive environment.

Magnetic scaffolds enhanced early cell attachment and spreading (Figure 2C). At days 3 and 7, polygonal osteoblast-like cells were evident on magnetic scaffolds, whereas cells on 0% MNP scaffolds appeared more elongated and aligned. After 28 days of culture in OM, magnetic scaffolds exhibited increased deposition of mineral-like nodules on the cell sheet surface (Figure 2D), indicating active biomineralisation promoted by MNPs.

Osteogenic differentiation of hBMSCs on PVA-Vat-MNP scaffolds was further evaluated through gene expression analysis. At day 7, scaffolds containing 6% MNP showed significantly higher expression of BMP2, RUNX2, COL1A1, and ALP (Figure 2E). By day 14, BMP2 and RUNX2 expression were further upregulated on scaffolds with higher MNP content, while COL1A1 and ALP levels on 6% MNP scaffolds did not significantly exceed those on 3% MNP scaffolds. Expression of OCN, a late-stage marker of biomineralisation and bone formation [27], was elevated on 6% MNP scaffolds at day 14, although no significant differences were observed among groups at earlier time points.

### 2.3. Magnetic Scaffolds Promote Angiogenesis In Vitro

**Figure 3:**
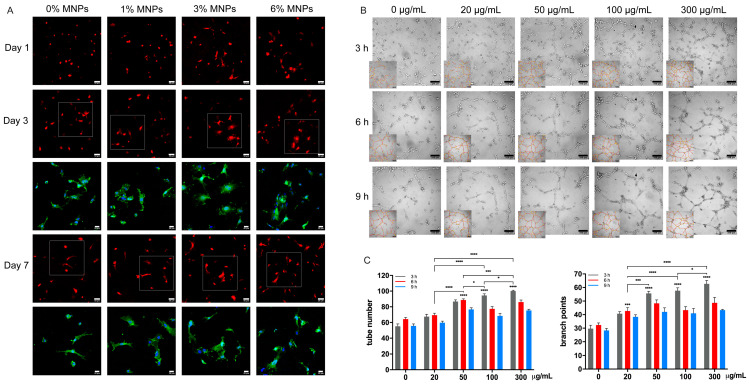
(**A**) Fluorescent images of RFP-tagged HUVECs cultured on PVA-Vat-MNP scaffolds for 1, 3 and 7 days. Actin staining within the highlighted regions (dashed boxes) showed the cell skeleton stained green and nuclei stained blue. (**B**) Representative images of HUVEC tube formation on Matrigel in the presence of bare MNPs at 0, 20, 50, 100 and 300 μg/mL for up to 9 h, with ramifications marked in red and branch points in yellow. (**C**) Quantification of tube numbers and branch points indicating more ramifications under magnetic stimulation at higher MNP concentration. Scale bars in A represent 50 μm and 20 μm; in panel (**B**), they represent 100 μm. * *p* < 0.05, *** *p* ≤ 0.001, and **** *p* ≤ 0.0001.

**Figure 4:**
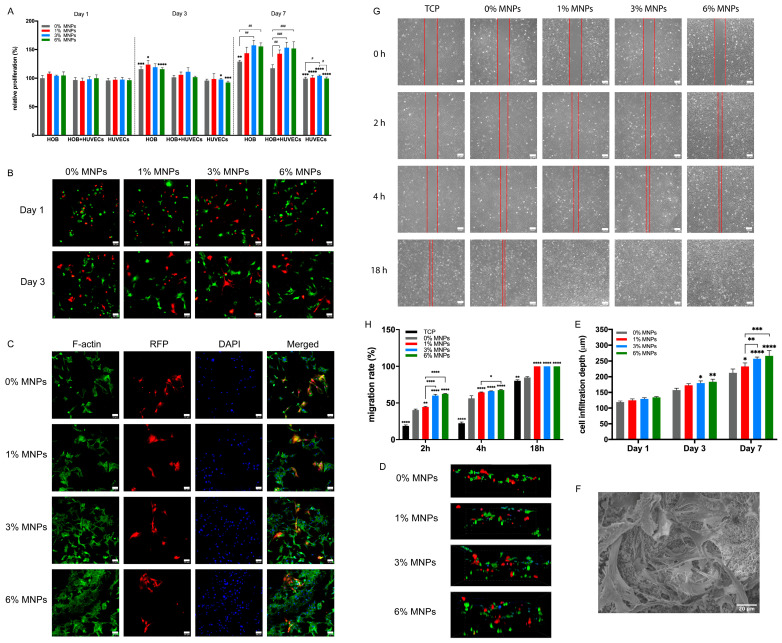
(**A**) Relative cell proliferation rate of the monoculture and coculture on PVA-Vat-MNP scaffolds for 1, 3 and 7 days, normalised to proliferation of HOBs on 0% MNP scaffolds at day 1. * indicates significant difference between monoculture and coculture in each group and # indicates significant difference between groups in the monoculture or coculture. (**B**) Fluorescent cell tracking images of coculture on scaffolds for 1 and 3 days, showing uniform distribution of HOBs (green) and HUVECs (red). (**C**) Actin staining of coculture on scaffolds at day 7. All cell skeletons were stained green, and all cell nuclei were stained blue, with RFP-tagged HUVECs shown in red. (**D**) Representative Z-stack CLSM images and (**E**) quantitative cell infiltration depth of coculture on scaffolds for 7 days. (**F**) Representative SEM image of cell infiltration through the interconnected porous structure of 3% MNP scaffold. (**G**) Representative images of migratory activity and (**H**) cell migration rate (%) of HUVECs cultured in CM from scaffold-treated HOBs. CM from HOBs cultured on tissue culture plastic (TCP) was used as control. Scale bars in panels (**B**,**C**) represent 50 μm; in panels (**F**,**G**), they represent 20 μm. ** *p* ≤ 0.01, *** *p* ≤ 0.001, and **** *p* ≤ 0.0001. ## *p* ≤ 0.01 and ### *p* ≤ 0.001.

Figure 3A illustrates that HUVECs adhered and spread well across all scaffolds, with cobblestone-like morphology and pronounced cytoplasmic extensions observed on scaffolds containing 3 and 6% MNPs by day 3. By day 7, some HUVECs exhibited an elongated morphology, suggesting progression towards tube formation along the matrix structure. Although the initial increase in surface cell density appeared modest (Figure 3A), alamarBlue assay results indicated a gradual and sustained proliferation of HUVECs in monoculture throughout the culture period (Figure 4A), suggesting potential cell migration into the macroporous scaffold structure had occurred.

**Figure 5:**
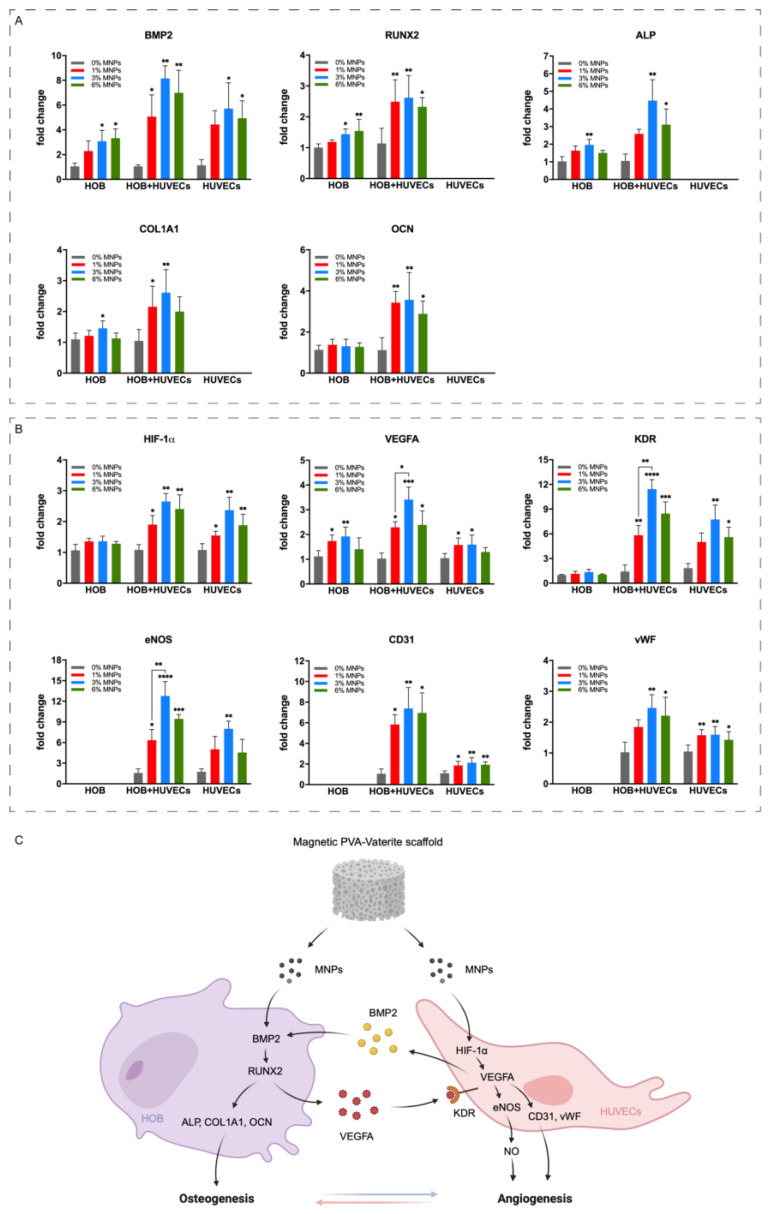
(**A**) Osteogenic and (**B**) angiogenetic gene expression of monoculture and coculture on PVA-Vat-MNP scaffolds for 7 days, normalised to gene expression on 0% MNP scaffolds in the monoculture and coculture, respectively. A significant increase in gene expression on scaffolds with a higher concentration of MNPs was observed in monoculture and coculture. (**C**) Schematic illustration of osteogenesis-angiogenesis coupling on PVA-Vat-MNP scaffolds via the BMP2/RUNX2 signalling pathway and the HIF-1α/VEGFA signalling pathway. * *p* < 0.05, ** *p* ≤ 0.01, *** *p* ≤ 0.001, and **** *p* ≤ 0.0001.

To further assess the angiogenic effect of MNPs, a tube formation assay was performed by culturing HUVECs with varying concentrations of bare MNPs. After 3 h on Matrigel, capillary-like structures were evident, where higher MNP levels promoted denser networks in tube-formation assays (Figure 3B). Quantitative analysis (Figure 3C) confirmed that higher MNP concentrations accelerated peak tube formation at 3 h, resulting in more tubes and branch points, thereby enhancing angiogenesis under magnetic stimulation. This observation aligns with increased angiogenic gene expression on magnetic scaffolds (Figure 5B). Following peak tube formation, all groups showed a decline in tube numbers and branch points, a common phenomenon observed in tube formation assays, reflecting the natural resolution process of vessel formation during angiogenesis [28].

### 2.4. Osteogenesis-Angiogenesis Coupling Capacity of PVA-Vat-MNP Scaffolds

As the primary bone-forming cells in intramembranous ossification during maxillofacial repair, HOBs were cocultured with HUVECs, either directly or indirectly, on PVA-Vat-MNP scaffolds to model bone formation. Fluorescent cell tracking images (Figure 4B) showed a uniform distribution of both cell types without noticeable clustering at early time points. Since HOBs proliferate faster than HUVECs [29], a higher density of polygonal HOBs was observed (Figure 4C), surrounding the clustered HUVECs at day 7. Higher MNP levels produced denser cell layers, aligning with the alamarBlue assay results (Figure 4A). At day 3, the coculture proliferation was intermediate between that of HOB and HUVEC monocultures. By day 7, a synergistic effect emerged: coculture proliferation exceeded that of HUVECs and approached the higher rate of HOBs, despite a 1:1 seeding ratio and low serum conditions that typically inhibit HOB growth. This suggests reciprocal crosstalk between the two cell types on the magnetic scaffolds. Cell infiltration (Figure 4D–F) confirmed active cell migration within the interconnected porous scaffold architecture, with greater infiltration observed in scaffolds containing more MNPs due to enhanced cell proliferation. An indirect wound healing assay using CM from scaffold-treated HOBs demonstrated accelerated HUVEC migration in groups with higher MNP concentrations (Figure 4G,H), showing increased migration at 2 and 4 h and complete wound closure by 18 h. MNP-stimulated HOB secretions enhanced HUVEC migration; however, no significant differences were observed between the 3% and 6% MNP groups in migration rate or infiltration depth.

### 2.5. Interaction Between Osteogenesis and Angiogenesis

Figure 5 showed that the osteogenic and angiogenic gene expressions were significantly upregulated in both monocultured and cocultured cells on magnetic scaffolds, which was more pronounced in the coculture system. Scaffolds containing 3% MNPs demonstrated the highest expression for most evaluated functional markers, with no significant further increase with 6% MNPs. Notably, in monocultured HOBs, BMP2 and RUNX2 expression peaked with 6% MNPs, mirroring the trend observed in hBMSCs (Figure 2E). While OCN expression remained unchanged in monocultured HOBs, it significantly increased in the presence of HUVECs, indicating the synergistic effect of osteogenesis and angiogenesis. Elevated CD31 and eNOS expression, critical for HUVEC adhesion and proliferation [30], aligns with alamarBlue results (Figure 4A) and suggests a positive effect on HUVECs likely due to endogenous VEGFA release from both cell types under MNP stimulation. The increased expression of KDR, a VEGFA receptor, correlates with increased VEGFA levels, highlighting enhanced angiogenic gene expression in coculture, particularly with MNPs up to 3%.

Osteogenesis and angiogenesis coupling involves complex cellular crosstalk [31]. The homogenous dispersion of nanoparticles throughout the 3D PVA-Vat-MNP scaffolds combined with their interconnected porous network structurally enhances the scaffold architecture [15] by increasing the overall surface area, roughness, and hydrophilicity. This improved topography promotes the adsorption of cell-adhesive proteins from biological fluids, thereby promoting superior cell proliferation and downstream signalling events [32]. Additionally, the incorporation of MNPs within the PVA matrix strengthens the scaffold mechanically, increasing its strength and stiffness (Figure 1C), consequently promoting greater cytoskeletal tension that accelerates cell cycle progression. These modified properties collectively foster superior cell-material interactions, creating a more favourable microenvironment essential for guiding the coupled bone and vessel formation. The integration of MNPs serves a dual function. Chemically, they facilitate calcium ion release by interrupting the electrostatic interactions between vaterite and PVA, thus accelerating its hydrolytic degradation, which supports enhanced biomineralisation activity [33]. Physically, as numerous studies have highlighted, MNPs can stimulate osteogenic differentiation via magneto-mechanical forces and potentially stimulate vascularisation [34]. In this study, we move beyond observing these effects individually and investigate the ability of PVA-Vat-MNP scaffolds to drive the synergistic coupling of osteogenesis and angiogenesis, emphasising critical cell-cell interactions and the activation of key signalling pathways under magnetic stimulation.

A 24 h burst release of calcium and iron initiates early cellular responses (Figure 1D). The rapid rise in extracellular calcium supports the activation and clustering of integrins [35], thereby strengthening cell-material adhesion, accelerating early cell attachment, and promoting the extensive filopodial spreading observed on the magnetic scaffolds at day 1 (Figure 2C). Additionally, this early calcium spike helps initiate the osteogenic commitment of hBMSCs, while the initial iron release provides a mild, transient redox signal through ROS generation that primes endothelial cells for angiogenic activity. Following the critical initial burst release, the tailored degradation kinetics of the scaffold ensure that both calcium and iron ions are maintained within optimal physiological windows. The sustained release of calcium ions effectively creates a mild hypercalcemic microenvironment (2 to 5 mM) that provides a potent biochemical stimulus for osteoblast proliferation, differentiation, and biomineralisation [36]. Concurrently, the controlled, low-dose release of iron ions in the µg/L preserves cellular integrity and gently drives the Fenton reaction to produce moderate levels of reactive oxygen species (ROS) and subsequently upregulate proangiogenic factors in endothelial cells [37].

During bone repair, osteoprogenitor cells and osteoblasts initiate the formation of new ossification centres within the defect site. These cells secrete proangiogenic factors that attract endothelial cells, promoting vascularisation. In turn, the newly formed blood vessels enhance the local delivery of osteogenic factors, promote osteoblast differentiation and facilitate bone regeneration. Additionally, these blood vessels may recruit mesenchymal stem cells, which differentiate into bone-forming cells, establishing a positive feedback loop that drives the coordinated processes of bone and vascular development [31].

The osteoblastic differentiation of hBMSCs was significantly enhanced on magnetic scaffolds, as evidenced by elevated ALP activity, improved cell adhesion, increased biomineralisation, and upregulated expression of osteogenic genes. Superparamagnetic iron oxide nanoparticles generate localised nanoscale magnetic fields, which can be further intensified by increasing MNP concentrations. These fields exert subtle magneto-mechanical forces that activate mechanosensitive ion channels on the cell membrane, including voltage-gated calcium channels and transient receptor potential channels [20]. The resulting ion influx works synergistically with the accelerated calcium ion release from the degrading magnetic scaffolds (Figure 1D), leading to a substantial increase in cytoplasmatic calcium levels. This strong intracellular signal activates the BMP2/RUNX2 pathway, a key regulator of bone formation, thereby promoting the expression of both early osteogenic markers (ALP, COL1A1) and the late-stage marker for osteoblastic maturation (OCN) [36,38]. This transcriptional increase in ALP is corroborated by the significantly higher alkaline phosphatase enzyme activity detected (Figure 2B), signifying the onset of early matrix maturation. As the culture period progresses, this matured matrix transforms into active mineralisation centres, driven by the late-stage upregulation of OCN. As a marker of terminal osteoblast differentiation, OCN binds calcium to regulate hydroxyapatite crystal formation, which is confirmed by the SEM images at day 28 (Figure 2D), revealing well-developed, cauliflower-like mineralised nodules deposited on the surface of magnetic scaffolds.

Interestingly, the highest MNP concentration (6%) appeared to limit further osteogenic progression in hBMSCs despite promoting the greatest cell proliferation and dense sheet formation. Rather than indicating cytotoxicity, this effect likely stems from density-dependent lineage commitment [39], a phenomenon where extensive cell-to-cell contact and resource competition favour proliferation over energy-intensive differentiation. Such conditions may explain the restricted maturation observed, suggesting a dose-dependent trade-off between proliferation and differentiation, consistent with earlier findings [40,41]. Moreover, the elevated RUNX2 expression at this concentration could paradoxically prolong the proliferative stage, given its regulatory role in balancing osteoblast proliferation and differentiation, contributing to the delayed and less pronounced increase in OCN expression [42].

HUVECs exhibited consistent proliferation across all scaffold types, although at a slower rate compared to hBMSCs. This difference may reflect the preference of HUVEC cells for smoother surfaces, combined with their inherent contact inhibition and relatively low proliferative capacity, which limits rapid expansion [43]. Enhanced cell adhesion, increased tube formation and upregulation of angiogenic genes under magnetic stimulation indicate improved angiogenesis, potentially driven by MNP-induced reactive oxygen species (ROS) [37]. MNP-derived iron induced ROS, which can inhibit prolyl hydroxylase activity and prevent proteasome degradation of HIF-1α [44]. Elevated HIF-1α subsequently promotes VEGFA expression and its receptor KDR, triggering downstream angiogenic markers such as eNOS, CD31 and vWF [45]. The upregulation of proangiogenic factors initiated early tubulogenesis, as demonstrated by the Matrigel tube formation assay (Figure 3B,C). The long-term functionality of vascularised bone grafts in vivo relies on the stabilisation and maturation of these newly formed capillary-like networks. The rigid interconnected 3D framework of the magnetic scaffolds provides a stable mechanical template that enables endothelial cells to align, form lumens, and progressively deposit a mature, native basement membrane. Meanwhile, the sustained MNP-induced secretion of VEGFA together with the reciprocal signalling between osteoprogenitors and endothelial cells provide a steady biochemical gradient that would prevent vessel collapse. However, this effect was dose-dependent, as at 6% MNP, no further increase in cell proliferation and angiogenic gene expression was observed. This suggests a dual role of ROS, where excessive iron-induced ROS overwhelms antioxidant defences, shifting from signalling to oxidative stress and ultimately impairing HUVEC proliferation and angiogenic activity [46].

The direct coculture of HOBs and HUVECs on magnetic scaffolds significantly enhanced cell proliferation, adhesion, and infiltration, suggesting its potential role in supporting intramembranous ossification during maxillofacial bone regeneration. Indirect coculture using CM from scaffold-treated HOBs also promoted HUVEC migration. These findings indicate that autocrine and paracrine signalling between HOBs and HUVECs, amplified by magnetic stimulation from MNPs, supports coordinated tissue responses. VEGFA, a key regulator of both osteogenesis and angiogenesis, is essential for intramembranous ossification; its deletion has been linked to impaired calvarial and mandibular developments [47,48]. During coculture on the magnetic scaffolds (Figure 5C), MNPs activated the BMP2/RUNX2 signalling pathway in HOBs through magneto-mechanical stimulation coupled with increased calcium ion release. This combination promoted osteoblast differentiation through autocrine and intracrine mechanisms and simultaneously elevated VEGF expression [31,49]. Secreted VEGFA then acted as a paracrine signal, activating KDRs on adjacent HUVECs, inducing endothelial proliferation and vascularisation through downstream gene expression [50,51]. Conversely, in HUVECs, the presence of MNPs and the associated release of free iron induced VEGFA expression via an ROS-dependent HIF-1α pathway and elevated endogenous BMP2 production, which in turn further enhanced osteogenic differentiation and matrix mineralisation in HOBs [17,52].

The expression of VEGFA and BMP2 was significantly higher on magnetic scaffolds, particularly in coculture, highlighting enhanced bilateral communication between osteogenesis and angiogenesis. Increasing MNP concentration amplified these effects; however, at 6%, this effect was diminished with a proliferative bottleneck limiting osteoblastic maturation and further angiogenic expression. Therefore, the 3% MNP concentration showed the optimal balance of effects. Chemically, the 3% formulation yields a controlled release of iron ions that maintains the ROS-HIF-1α signalling axis to maximise angiogenic signalling without overwhelming cellular antioxidant defences. Physically, the 3% scaffold provides sufficient mechanical support and optimal magneto-mechanical stimulation that robustly activates osteogenic differentiation. This formulation precisely calibrates these physicochemical parameters and achieves the ideal equilibrium required for orchestrating the complex interplay of bone and vessel regeneration.

The clinical potential of this magnetic platform lies in its ability to overcome key limitations of current passive scaffolds by offering a cell-free strategy that bypasses donor site morbidity and complex cell handling. Unlike traditional scaffolds relying on the uncontrolled release of pre-loaded growth factors, this system enables active, non-invasive post-implantation control, allowing clinicians to dynamically guide and synchronise osteogenesis and angiogenesis to address the critical challenge of graft vascularisation. Ultimately, validating these findings through rigorous preclinical studies in large animal models with critical-sized defects will be essential to establish the safety and efficacy required to bring this regenerative technology to a successful clinical translation.

## 3. Conclusions

This study demonstrates that 3D porous PVA-Vat-MNP scaffolds effectively promote osteogenic differentiation in hBMSCs while enhancing vascularisation in HUVECs, establishing a strong synergistic interaction between osteogenesis and angiogenesis in coculture systems. However, these effects were both dose-dependent and cell-type specific, underscoring the importance of optimising MNP concentrations to achieve maximum therapeutic benefit. Importantly, these findings provide a mechanistic foundation and an optimised formulation to guide future preclinical in vivo studies, positioning PVA-Vat-MNP scaffolds as a promising platform for bone tissue engineering by enabling coordinated bone and vascular regeneration.

## 4. Materials and Methods

### 4.1. Preparation and Characterisation of PVA-Vat-MNP Scaffolds

As described in a previous study [15], MNPs were synthesised by a solvothermal method [53], and vaterite particles were fabricated by a fast precipitation method [54] with casein as an additive. Briefly, MNPs (0, 1, 3 and 6% of total weight) were suspended homogeneously in 10% (*w*/*v*) PVA solution, then mixed with vaterite particles (PVA:vaterite = 40:60). The MNP concentrations were primarily selected based on data from prior studies, which typically observed positive effects of MNPs up to a maximum of 10 wt%, while also considering the feasibility of maintaining suitable composite paste viscosities for scaffold fabrication [49]. The obtained paste was cast into cylindrical moulds, followed by two freeze-thaw (FT) cycles. Specifically, each cycle consisted of freezing at −80 °C for one hour, freeze-drying for 24 h and thawing for 24 h at room temperature. During the initial rapid freezing at −80 °C, the uniform nucleation of ice crystals induces immediate phase separation, forcing the PVA chains into densely packed, polymer-rich regions and initiating extensive hydrogen bonding and microcrystallite formation. Subsequently, the direct sublimation of these interconnected ice crystals during freeze-drying leaves behind a continuous void space, resulting in a highly interconnected macroporous architecture. Finally, the thawing phase allows for polymer chain relaxation and further stabilisation. Compressive strength and modulus, degradation, scanning electron microscope (SEM) and microcomputed tomography (μCT) images of scaffolds were acquired in our previous study [15]. The cumulative release of calcium and iron ions in deionised water was detected by inductively coupled plasma mass spectrometry (ICP-MS, NexION 5000, PerkinElmer, Shelton, CT, USA).

### 4.2. Cell Culture

Primary human bone mesenchymal stem cells (hBMSCs, PromoCell, Heidelberg, Germany) were cultured in Dulbecco’s modified Eagle’s medium (DMEM, Sigma, St. Louis, MO, USA) supplemented with 10% fetal bovine serum (FBS, Sigma) and 1% penicillin-streptomycin (Sigma). Primary human osteoblasts (HOBs, PromoCell) were cultured in DMEM supplemented with 10% FBS, 1% penicillin-streptomycin, 2% 4-(2-hydroxyethyl)-1-piperazineethane sulfonic acid (HEPES, Sigma), 1% minimal essential medium, 20 mM L-glutamine, and 0.15 mg/mL L-ascorbic acid powder. Red fluorescent protein (RFP)-tagged human umbilical vein endothelial cells (HUVECs, 2Bscientific, Kirtlington, Oxfordshire, UK) were cultured in endothelial cell growth medium 2 with Supplement Mix (EGM2, PromoCell). Cells were seeded at a density of 5 × 10^4^ cells/scaffold.

### 4.3. Cell Viability and Attachment

Cell viability was evaluated by alamarBlue assay for up to 28 days. Aliquots of the reacted solution were transferred to a fresh 96-well plate (*n* = 4) prior to fluorescence measurement. Cell attachment on the scaffolds at days 1, 3 and 7 was visualised by actin staining with phalloidin (Alexa Fluor 488, Thermo Fisher Scientific, Waltham, MA, USA) and 4,6-diamidino-2-phenylindole (DAPI, Alexa Fluor, Thermo Fisher Scientific) using a confocal laser scanning microscope (CLSM, LSM 980, ZEISS, Oberkochen, Germany). The use of CLSM provided optical sectioning and mitigated background scattering, while the dark colouration of magnetic scaffolds reduced optical noise and enhanced the contrast and signal-to-noise ratio for the fluorescently labelled cells.

### 4.4. Scanning Electron Microscope (SEM)

hBMSCs were cultured in osteoblastic differentiation medium (OM) containing 100 nM dexamethasone, 10 mM β-glycerophosphate and 0.15 mg/mL ascorbic acid for 28 days and then fixed and observed under SEM (JCM-6000plus, JEOL, Tokyo, Japan).

### 4.5. Alkaline Phosphatase (ALP) Activity

After culturing in stem cell growth medium (GM) or OM for up to 28 days, the ALP production in cell lysates of hBMSCs was evaluated by SIGMAFAST™ p-Nitrophenyl Phosphate Tablet Sets (Sigma) according to the manufacturer’s protocol. The ALP activity was normalised to the corresponding total protein production in cell lysates, measured by Quick Start™ Bradford Protein Assay Kit (Bio-Rad, Hercules, CA, USA) following the manufacturer’s protocol.

### 4.6. Capillary Tube Formation Assay

The angiogenic potential of bare MNPs was assessed by the tube formation assay using Matrigel Basement Membrane Matrix (Corning, Steuben County, NY, USA). 1.5 × 10^4^ HUVECs were seeded on Matrigel (50 μL/well) in a 96-well plate and cultured in EGM2 containing 0, 20, 50, 100 and 300 μg/mL MNPs. These specific concentrations were selected based on previous studies on the dose-dependent effects of MNPs [40], with the lower concentrations mimicking physiological accumulation of MNPs and iron ions within a confined, low-fluid-turnover microenvironment of a maxillofacial bone defect post-implantation and the 300 µg/mL dose serving as an upper-limit boundary to evaluate dose-dependent saturation. The well centre was observed under a light microscope (Olympus IX51, Tokyo, Japan) at different time points and imaged by a connected digital camera (Colourview II, Olympus). The tube number and branch points were quantified using Image J 2.1.0.

### 4.7. Coculture

HOBs were labelled with CellTracker™ Green CMFDA (Invitrogen, Carlsbad, CA, USA), while HUVECs were RFP labelled in coculture. HOBs and HUVECs were seeded on the PVA-Vat-MNP scaffolds at a ratio of 1:1. A mixture of HOB culture medium and EGM2 at a ratio of 1:1 was used for coculture. HOBs and HUVECs were monocultured on the scaffolds at the same density as the control. The cell viability was measured using alamarBlue assay, and cell morphology was observed under CLSM and SEM.

### 4.8. Wound Healing Assay

HUVECs were seeded in 6-well plates at a density of 2 × 10^4^ cells/cm^2^ and maintained in EGM2 until reaching full confluence. A scratch was created using a 200 μL pipette tip, followed by two PBS washes to remove detached cells. The remaining cells were then cultured in conditioned medium (CM) collected from HOBs grown on scaffolds for 3 days to assess the paracrine influence of scaffold-treated HOBs on HUVECs. Cell migration was monitored under a light microscope and captured using a digital camera at various time points. The migration rate was calculated as

Migration rate (%) = (D_0_ − D_t_)/D_0_ × 100%

D_0_: the initial wound distance.

D_t_: the remaining wound distance at each time point.

### 4.9. Quantitative Real-Time Polymerase Chain Reaction (qRT-PCR)

The RNA was extracted using Tri Reagent™ (Invitrogen) following a modified protocol [55] to improve RNA purity and subsequently, reverse transcribed to cDNA using a High-Capacity cDNA Reverse Transcription Kit (Applied Biosystems, Foster City, CA, USA). Predesigned human primers (Table 1) were used to quantify osteogenic genes—alkaline phosphatase (ALP), runt-related transcription factor 2 (RUNX2), bone morphogenetic protein-2 (BMP2), collagen 1 alpha 1 (COL1A1) and osteocalcin (OCN)—as well as angiogenic genes, including hypoxia-inducible factor 1-alpha (HIF-1α), kinase insert domain receptor (KDR), vascular endothelial growth factor A (VEGFA), endothelial nitric oxide synthase (eNOS), platelet endothelial cell adhesion molecule (PECAM1 or CD31), and von Willebrand factor (vWF). Ribosomal protein L13a (RPL13A) served as the housekeeping gene. Gene expressions were analysed by qRT-PCR using PowerUp™ SYBR™ Green Master Mix (Applied Biosystems), normalised to the (RPL13A) and calculated using the ΔΔCT method.

### 4.10. Statistical Analysis

All proliferation and differentiation experiments were conducted in quadruplicate, whereas capillary tube formation and wound healing assays were performed in triplicate. These sample sizes were selected to ensure sufficient statistical power to detect effects associated with the magnetic scaffolds, while the use of well-characterised, commercially sourced primary cells minimises the inherent donor-to-donor biological variability in primary cultures. Data are presented as mean ± standard deviation (SD). One-way and two-way ANOVA with post hoc Tukey analysis were performed using GraphPad Prism 9 (* *p* < 0.05, ** *p* ≤ 0.01, *** *p* ≤ 0.001, and **** *p* ≤ 0.0001).

## Figures and Tables

**Table 1:** Primer sequences used for qRT-PCR.

Gene	Forward (5′-3′)	Reverse (5′-3′)
RPL13A	GGATGGTGGTTCCTGCTG	TGGTACTTCCAGCCAACCTC
ALP	AACACCACCCAGGGGAAC	TGGCATGGTTCACTCTCGT
RUNX2	AATGGTTAATCTCCGCAGGTC	TTCAGATAGAACTTGTACCCTCTGTT
BMP2	TCCACCATGAAGAATCTTTG	TAATTCGGTGATGGAAACTG
COL1A1	GCTATGATGAGAAATCAACCG	TCATCTCCATTCTTTCCAGG
OCN	CCAGGCGCTACCTGTATCAA	CCTGAAAGCCGATGTGGTC
HIF-1α	AAAATCTCATCCAAGAAGCC	AATGTTCCAATTCCTACTGC
VEGFA	GGAGGCAGAGAAAAGAGAAAGTGT	TAAGAGAGCAAGAGAGAGCAAAAGA
KDR	GTACATAGTTGTCGTTGTA	TCAATCCCCACATTTAGTTC
eNOS	CAACCCCAAGACCTACG	CGCAGACAAACATGTGG
CD31	ACGTGCAGTACACGGAAGTT	GGAGCCTTCCGTTCTAGAGT
vWF	AGCCCATTTGCTGAGCCTTG	CCTGGCACCATGCATTTCTG

## Data Availability

The original contributions presented in the study are included in the article. Further inquiries can be directed to the corresponding author.
